# Current research on public perceptions of nanotechnology

**DOI:** 10.3134/ehtj.10.164

**Published:** 2010-09-25

**Authors:** J Besley

**Affiliations:** School of Journalism and Mass Communications, University of South Carolina, Columbia, SC, USA

## Abstract

This review explores research on public perceptions of nanotechnology. It highlights a recurring emphasis on some researchers’ expectations that there will be a meaningful relationship between awareness of nanotechnology and positive views about nanotechnology. The review, however, also notes that this emphasis is tacitly and explicitly rejected by a range of multivariate studies that emphasize the key roles of non-awareness variables, such as, trust, general views about science, and overall worldview. The review concludes with a discussion of likely future research directions, including the expectation that social scientists will continue to focus on nanotechnology as a unique opportunity to study how individuals assess risk in the context of relatively low levels of knowledge.

## Introduction

As this review shows, a number of scholars in social science disciplines, such as political science and science communication, have turned their attention to exploring how individuals perceive nanotechnology's risks and benefits. Research in this area continues to provide a unique opportunity to track risk perceptions, whereas nanotechnology discussions are just beginning to appear in the public sphere. Many of those involved in this research, and cited below, had previously studied emerging technology areas, such as nuclear energy and agricultural biotechnology, and saw the opportunity to help decision makers avoid some of the communication missteps that proponents of previous technologies had committed. Technology advocates often point to these communication failures as the cause of unwarranted public health and environmental concerns.^1^ At the academic level, the emergence of nanotechnology as a potential subject of social research also corresponded with more general discussions within academia and government in both Europe and North America about the value of ‘upstream’ public involvement in science decision making.^2^–^4^

The current review describes research on nanotechnology perceptions, with an emphasis on both studies focused on basic nanotechnology awareness as well as more theoretically guided work that focuses on understanding the dynamics underlying nanotechnology attitude formation and expression. In doing so, it seeks to identify challenges in the literature, particularly areas where scholars have failed to adequately draw on previous literature in developing their research. It finishes with a brief discussion of the likely future direction of work in this area. The articles noted were found based on initial Web of Science searches for: ‘nano*’ AND (‘opinion*’ OR ‘survey*’ OR ‘experiment*’). The cited references for these articles were then examined for additional relevant studies. The search also highlighted several media content analyses, which are also summarized briefly below to provide context for the opinion-oriented research. As a review of public perceptions, only studies where an attempt was made to obtain a representative sample of a population (or sub-population) are included. Although many of the studies use random digit dialing, several use mail or online surveys. This excludes, for example, experimental studies using non-probability samples^5^, ^6^ or volunteer surveys.^7^ It also excludes qualitative studies that explore how individuals shape their views about nanotechnology and associated actors through discussion, although these generally support the results described below.^8–10^ Because this review represents secondary research, no institutional review board approval was sought for this review.

Nanotechnology involves ‘the understanding and control of matter at the dimensions of roughly 1–100nm where unique phenomena enable novel applications’.^11^ This definition emphasizes that nanotechnology is not a specific product but is, rather, a scale at which scientists or companies may manipulate or produce objects. The ability to work at this level opens up opportunities for advances in key economic fields, such as electronics, materials and pharmaceuticals, as well as a range of consumer product areas such as cosmetics. As of mid 2010, the Project on Emerging Nanotechnologies (PEN), a project of the Woodrow Wilson International Center for Scholars and the Pew Charitable Trusts, had identified more than 1000 consumer products that manufacturers have specifically said to make use of nanotechnology, with more applications becoming commercially available every month.^12^ Although one study has questioned the validity of the PEN database,^13^ it is clear that many nanotechnology products are currently on the market.

Government agencies responsible for science and economic development in many countries have identified nanotechnology as a key area of strategic importance and have devoted substantial resources toward basic and applied research.^14–16^ Several social science-based projects have also received funding. The PEN initiative has funded a number of surveys, and many of the studies described below are the result of a large National Science Foundation (NSF) initiative to create the Center for Nanotechnology in Society at Arizona State University^17–23^ and the Center for Nanotechnology in Society at the University of California, Santa Barbara. The NSF has also provided funding to social scientists at other universities, including the University of South Carolina,^8, 23, 24^ Cornell University,^25, 26^ North Carolina State University,^6, 27, 28^ Yale University^29^ and Rice University.30 Even studies focused on Britain and Europe report receiving some NSF support.^9, 31^ Funding has also been provided for humanities-oriented work in areas such as philosophy and history but these areas will not be discussed here. Nevertheless, critics have often argued that funders are devoting too little funding to assess the potential health, environmental and social consequences of nanotechnology.^32, 33^ This tension between economic, social and ethical concerns has been captured in recent research on media coverage of nanotechnology.

### Media content finding: focus on progress

In addition to the survey research described below, a number of systematic assessments of media content have also focused on nanotechnology, starting with a handful of studies in 2005. Building on similar work about previous emerging technologies,^34–36^ the nanotechnology content analyses show that most coverage tends to focus on technological process and, only rarely, health and environmental risks or ethical concerns. Health and risk content appears to have become more common over time but, overall, coverage of any aspect of nanotechnology has continued to remain relatively rare.^23, 37–41^ These initial content analyses looked at the United States^23^,^37, 38^ and the United Kingdom.^23, 38–40^ More recent studies have looked in more detail at subissues such as nanoparticle safety,^40^ the emergence of more increased regulatory focus,^41^ economic coverage of nanotechnology, as well as coverage in smaller countries such as Denmark.^42^ Some recent studies have also relied on interviews with journalists who cover nanotechnology to emphasize the challenges of communicating uncertain science appropriately.^43, 44^ Given the relative paucity of coverage, however, there is no evidence that the news media are driving the debate about nanotechnology.

### Survey research finding: low knowledge

It is consistent with the low levels of media coverage and it should perhaps come as little surprise that the earliest and most consistent finding of nanotechnology survey research is that the public does not know much about nanotechnology. For example, one attempt by Satterfield *et al*.^45^ to synthesize the survey work up to 2008 shows that about half of respondents in the reported studies said they had no familiarity with nanotechnology. Annual telephone surveys conducted for the Woodrow Wilson center starting in 2006, some of which were included in the summary study, report that in 2006 and 2007, 42% of respondents (n = ~ 1000 in all years) said they had heard nothing about nanotechnology. This number rose to 49% in the 2008 survey and then backed down to 37% in 2009. At the same time, the number of people who said they heard ‘a lot’ about nanotechnology hovered between 24 and 31% (ref. 46, see also online Supplementary material in ref. 47). Similar results from face-to- face surveys in the United Kingdom^48^ and telephone surveys in Canada^49^ and Japan^50^ were included in the summary study. An additional convenience sample study of (primarily) young people^26^ and more recent online studies from Germany^51^ and France^52^ also found similar results in those countries. A broader European study, although not specifically asking about nanotechnology awareness, found that 53% of Europeans sampled in a 2004 survey said that they did not know enough to answer a question about whether nanotechnology ‘will improve (their) way of life in the next 20 years (29%)’ or whether it would have no effect (12%) or whether it will make things worse (6%).^31^

One noteworthy aspect of the research underlying the consensus that the public know little about nanotechnology is that most of the data focus on respondents’ self-reported level of awareness. Few studies include tests specifically meant to assess knowledge (that is, true/false tests). When administered, these tests indicate that, on balance, respondents generally understand that nanotechnology is an economic issue, is invisible to the naked eye and involves the modification of materials.^18, 20, 28^

### Survey research finding: benefits outweigh the risks

**Figure 1:**
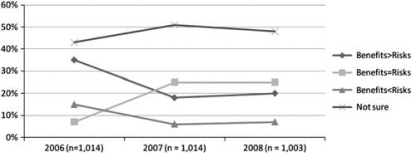
Initial impression of nanotechnology benefits versus risks from the Project on Emerging Nanotechnology as adapted from surveys collected by Peter D Hart Associates.^62, 79^

Beyond reports of low knowledge levels, the second most common survey feature is the finding that when people have an opinion (and an average of about half of those surveyed did not), they see more promise than peril in nanotechnology. ^46^ Most of this research (for example, refs 28, 29, 31, 48, 49, 51–53) involves asking survey participants directly whether they think nanotechnology will be, on balance, good or bad. For example, the Peter D Hart Associates research for the Woodrow Wilson^46^ center asks respondents to indicate whether ‘benefits will outweigh the risks,’ ‘benefits will about equal the risks,’ ‘risk will outweigh the benefits’ or whether the respondent is ‘not sure’ (Figure 1).

In contrast, telephone surveys by Scheufele and colleagues ^17, 18, 20–22^ as well as mail surveys by Siegrist and his colleagues^54^,^55^ ask multiple questions about specific potential risks of nanotechnology, followed by multiple questions about the potential benefits of nanotechnology. The goal in doing so was to ensure that the survey assessed the range of potential risks and benefits. One mail survey-based study used a hybrid approach asking for a direct weighting of relative risks and benefits, as well as questions specifically about perceived health and environmental risks.^25^ Although the different approaches to measurement still point to more positive than negative attitudes about nanotechnology, risks and benefits may also interact to amplify or attenuate the impact of such perceptions on willingness to accept nanotechnology.^30^

### Survey research: relationship between awareness and attitude

Beyond simply describing nanotechnology awareness and perceived risks and benefits, most academic studies of nanotechnology opinion attempt to test the degree to which specific factors are driving opinion. As might be expected, the most common relationship assessed is the one between awareness and perceived risks and benefits. This focus on the relationship between knowledge and attitudes toward science has been the subject of substantial research for many years. Although scientists often appear to believe that, if people just knew more, they would have more positive attitudes toward its products,^56, 57^ academic research on this topic tends to show that that knowledge has relatively limited impact on attitudes toward emerging technologies. ^58, 59^ Science communication scholars have come to use the term ‘deficit model’ as a term-of-art in critiques of actors—usually scientists—who expect that increased scientific knowledge will inevitably lead to increased public acceptance of science.^60, 61^

The difficulty with critiquing the argument that knowledge is associated with attitudes is that the relationship sometimes exists.^58^ Indeed, a meta-analysis by Satterfield *et al*.^45^Fwhich focuses on similar themes to those described here but with a greater focus on the meta-analysis, less emphasis on critical review of findings from multivariate models, less emphasis on measurement and includes several different studies—confirms that that the available nanotechnology data suggest that increased self-reported awareness is associated with marginally more positive views. Online surveys from Germany^51^ and France,^52^ which were not included in Satterfield further, appear to suggest that awareness is associated with more positive views about nanotechnology after demographic controls and several additional explanatory variables (see Table 1). However, the studies that use the more elaborate test-based measures of knowledge (rather than self-reported awareness) find that basic science literacy rather than nanotechnology specific literacy is the more important predictor of positive views about nanotechnology,^18, 20–22^ although it is impossible to compare these studies directly because the latter studies focus on dependent variables, such as support for nanotechnology policies, rather than risks and benefits. One mail survey-based study that uses both an awareness self-report and a short, general science literacy quiz finds that although awareness is associated with lower risk perceptions, general science knowledge is not. This study, however, uses only a limited regional sample.^25^

**Table 1:** Key variables in multivariate analyses of public opinion data about nanotechnology

*Study*	*Survey year*	*Risk/ benefits*	*Self-report aware*	*Knowl. (general/nano)*	*Trust*	*Religion*	*View of science/ science media use*	*Enviro values*	*Survey mode*
Cobb and Macoubrie^27^	2004		Sig.	NS	Sig.		Sig. (view of sci)		Tel.
Lee *et al.*^18^^a,*^	2004	DV	NS	Sig.			Sig. (both)		Tel.
Gaskell *et al.*^31^	2004	DV			Sig.		Sig. (view of sci.)	Sig.	Face
Scheufele and Lewenstein^20^^a,*^	2004	Sig.	NS				Sig. (sci. media)		Tel.
Brossard *et al*.^17^^*a*,*^	2004	Sig.		NS (nano)		Sig.	Sig. (sci. media)		Tel.
Siegrist *et al.*^55^^b^	Not report	DV			Sig.		Sig. (view of sci.)		Mail
Cook and Fairweather^67^^c^	2006	Sig.				Sig.			Mail
McComas *et al.*^25^^d^	2006	DV	Sig.	NS (general	NS^d^	Sig.	NS (sci. media)		Mail
Kahan *et al.*^*29*^	2006	DV	Sig.						Online
Stamfli *et al.*^54^^b^	2006	Sig.			Sig.		Sig. (view of sci.)		Mail
Scheufele *et al.*^47^^**,e^	2007			Sig. (nano)	NS^d^	Sig.			Tel.
Cacciatore *et al.* (online first)^22**,a^	2007	Sig.		Sig. (nano)		NS	NS (sci. newspaper, sci. web),		Tel.
					.	.	Sig. (sci. TV)		
Ho *et al.*^21**,^a^^	2007	Sig.		NS (nano)	Sig.	Sig	Sig. (both)		Tel.
Vandermoere *et al.* (online first)^52^^f^	2008		Sig.		Sig		Sig. (view of sci.).	Sig	Online
Vandermoere *et al*^51^	2008	DV	Sig.			Sig.	Sig. (view of sci.).	Sig	Online

Abbreviations: DV, dependent variable in study, Sig.; variable was significant in final model; NS, variable was not significant in final model.

a DV=for support for nanotechnology funding.

b DV=willingness to buy nanotechnology related food product.

c DV=willingness to buy nanotechnology beef or lamb.

d Interpersonal fairness.

e DV=moral acceptability.

f DV=support for nanotechnology food packaging.

*, ** Used the same survey data. Tel.=telephone.

### Survey research: experimental studies

Several survey studies have embedded a knowledge-building or issue-framing experiment into the design of the survey itself. For example, an early phone survey-based experiment gave respondents a description of nanotechnology framed in one of 10 different ways (for example, focus on health benefits, focus on health risks and so on) and found that nanotechnology explanations that focused only on risks or only on benefits change the balance of perceived risks and benefits.^27^ Smiley Smith *et al*.^53^ analyzed a more straightforward pre-post survey-based experiment built into the 2006 Project for Emerging Nanotechnologies data in the United States^62^ and found that, when given a few paragraphs of basic information, some types of respondents (that is, men, those with more than a high school education and Republicans) were more likely to switch from saying that they did not know how they felt about nanotechnology to saying they believed that the benefits outweighed the risks. Other work, however, directly challenges this perspective by showing that increased information likely works by increasing the degree to which respondents draw on their preexisting cultural frameworks rather than the new information itself.^29^ On a different question, Siegrist^63^ embedded questions about food into one of their mail surveys and found that using nanotechnology to give food health benefits made the food less attractive than unmodified food.

### Survey research: other explanatory variables

Trust has emerged as a standard variable in the risk communication literature^64^ and it has therefore been an important element of nanotechnology research as well. Some of the first published random sample-based surveys on nanotechnology emphasized that trust in business leaders^28^ and trust in scientists^18^,^21^ represent important predictors of views about nanotechnology. A later study similarly focused on general social trust,^55^ whereas others pointed to confidence in federal regulators and business leaders^53^ or a composite measure of business and scientific authorities.^31^ At a measurement level, the most comprehensive examination of trust and nanotechnology can be found in recent research by Siegrist^54^, aimed at understanding the willingness to buy nanotechnology-related foods. This study used a mail survey with a relatively small sample and found that trust in scientists and government regulators, as well as trust in private sector food actors, are associated with perceived benefits of nanotechnology food. Trust in private sector actors is also associated with lower perceived risk.^54^ Another mail survey-based study looked at the related concept of fairness, including the degree to which respondents felt scientists were interpersonally respectful and polite, but did not find relationships between this variable and views about nanotechnology.^25^

Several research projects have also explored religion as an explanation for nanotechnology views. This work is partially premised on work related to emerging medical technologies such as those associated with stem cell research.^65^ Such research emphasizes that religion provides one of several key predispositions or orientations that underlie attitudes about technologies.^66^ Although the German survey analysis reports little evidence that religiosity affects views about nanotechnology, ^51^ one US study showed that religiosity is associated with overall lower support for nanotechnology funding; it also appeared to interact with knowledge to keep nanotechnology support down among religious individuals who also have relatively high levels of knowledge^17^ (see also refs 21, 22). Religious importance has also been used as a basic control variable and found to be significant predictor of support for science authority^25^ and food perceptions.^67^

The other main predictors of nanotechnology explored in the literature are overall attitudes toward science and views about the environment. As might be expected, given low levels of specific knowledge about nanotechnology, the general ‘attitude toward science’ variable has proven to be one of the most consistently significant predictors of views about nanotechnology.^18, 21, 28, 31, 51, 52^ Science media use, inasmuch as it also represents an underlying interest in science, has similarly been found to have a significant role in some studies.^17, 18, 20, 47^ The positive relationship between general support for, or interest in, science is contrasted by a negative relationship between environmental values and positive views about nanotechnology.^31, 51, 52^

A mail survey study from New Zealand that looked at self reported intention to purchase lamb or beef that had been genetically modified using nanotechnology^67^ found that a number of variables associated with Theory of Planned Behavior, including self-identity, attitude toward such modifications and perceived norms,^68, 69^ were significant predictors of potential buying intentions.^67^ The study did not, however, include any of the other standard variables, such as trust, knowledge or views about the religion or the environment.

Table 1 summarizes the multivariate studies that have looked at views about nanotechnology.

Demographics have not generally been a key element of discussion about nanotechnology perceptions but some specific variables are consistently significant predictors of views about risk.^70^ In the studies reviewed here, men,^18, 20, 25, 31, 51, 53, 55, 68^ older respondents,^31, 53^ Whites,^28, 47^ those with relatively higher levels of education,^18, 28, 31, 47, 53, 67^ those with relatively higher levels of income^18^,^53, 67^ and conservatives^31^,^25^ sometimes appear to be more positive about nanotechnology in the available multivariate analyses.

### Survey research: experts' views

A small number of studies have explored what nanotechnology scientists think about the risks and benefits of nanotechnology. Two of the surveys drew on an attempted census of US main authors who published on nanotechnology in ISI-referenced journals,^19, 71, 23^ whereas the third attempted a census of scientists at a specific European conference.^55^ Two of these articles included comparison data from public opinion surveys.^19, 55^ The two US-focused surveys, one of which resulted in two separate studies,^23, 71^ find that scientists see a range of benefits for nanotechnology, particularly in the areas of health and the environment, but that they are also concerned about health and environmental impacts,^23^ perhaps even more so than the public.^19^ The European study suggested that the public makes risk judgments based on a combination of social trust, perceived risk benefits and overall views about technology, whereas only social trust matters have a part in scientists’ risk judgments.^55^

### Conclusions and next steps

Given the number of researchers currently engaged in the field, as well as the fact that these respondents come from a range of disciplines, the social scientific literature related to nanotechnology perceptions is likely to continue to expand for the foreseeable future. Few social scientists associated with nanotechnology research would likely admit to hoping that public opinion about the subject follows the trajectory of previous emerging technologies such as agricultural biotechnology or nuclear energy and becomes an object of social division. Nevertheless, the survey work described can provide the field baseline findings against which to compare any future changes in the public perceptions, should nanotechnology emerge as a contentious public issue. Even if few questions about nanotechnology surface, the study of nanotechnology has provided a unique opportunity to test the key role that variables such as trust, cultural worldviews and religion have in shaping views about technology. Further, showing that scientific knowledge is rarely a key predictor of attitudes about nanotechnology gives social scientists the opportunity to demonstrate their expertise to research scientists and professionals more directly involved in scientific research.

Rather than focusing on simply educating members of the public, for example, Nisbet and Scheufele^72^ have argued that scientists need to work with communication experts to frame emerging science in ways that resonate with citizens’ existing worldviews. This perspective has also contributed to discussions about how to best address skepticism over climate change.^73^ Kahan, whose work points in a similar direction,^29, 66^ has recently explored the usefulness of this approach in the context of the emerging area of synthetic biology in working papers.^74, 75^ Nisbet and Scheufele^72^ and others^25^ have also argued that the primacy of variables such as trust suggests the need for scientists to devote substantial resources to honest and respectful engagement with the public.

Even if no public debate emerges, additional research may also benefit from focusing on specific rather than general aspects of nanotechnology. In the area of biotechnology, for example, it became apparent over time that the public had different views about biotechnology in animals compared with plants.^76^ The expert surveys described above,^19, 23^ the experimental work by Cobb^27^ and some of the qualitative work not reviewed here^9, 10^ explored questions about specific nanotechnology applications. However, the work by Siegrist and his colleagues^54^ on food issues is the most specific work in this area. Performing valid research in the context of probability surveys, however, remains difficult given the low levels of general knowledge. Another potentially promising path to better understand public opinion about nanotechnology is to design studies that enable direct comparisons of views about nanotechnology and other technologies, an approach that has been part of only a few of the studies described above.^25, 30^

Survey mode, although addressed here in passing, may have a role in survey quality. Whereas online and mail surveys, if done appropriately, can often enable more detailed measurement at lower cost,^77^ they can also suffer from low response rates, and substantial efforts are required to demonstrate that the included sample adequately represents the target population.^78^ Whereas the Kahan nanotechnology data^29^ appears to satisfy this requirement, the French and German online panels appear to have used a less rigorous sampling methodology. Future research might explore whether survey mode has an impact on perceptions of emerging technology.
